# 

**Published:** 1867-02

**Authors:** 


### CONTENTS FOR JANUARY.

ORIGINAL CONTRIBUTIONS.

On Dislocations of the Shoulder, reduced by Manipulation. By D. G. Rush, M.D., of Chicago, ---- 49

Treatment of Malarial Disease. By W. Anderson, M.D., of Benjaminville, Ill., ---- 53

"Connubimur." By T. O. Edwards, M.D., of Lancaster, Ohio, ---- 56

SELECTED ARTICLES, ---- 60

Suggestions for the Treatment of Cholera. By Arthur Ernest Sansom, M.D.—Climacteric Menorrhagia. Extract from a Lecture Delivered by Prof. B. F. Barker, at Bellevue Medical College, Dec. 11, 1866—East Indian Quinine—Deformities of the Nose. Operation to Repair a Broken Down Septum Narium. By Chas. F. Taylor, M.D., of New York—Case of Fracture of the Hip-Joint, with other Severe Injuries, Treated without Splints. By Theodore W. Fisher, M.D., Boston—Prof. O. W. Holmes' Microscope—Dr. Henry Bennett on the Curability of Consumption, etc. Mr. Lockhart Clarke's Investigations of the State of the Spinal Cord in Tetanus, etc.—A Morgue in New Orleans—Extract from a Paris letter, dated November, 1866—Scabies. Paraffine.

PROCEEDINGS OF MEDICAL SOCIETIES.

Chicago Medical Society, ---- 83

Singular Parasite—Laryngeal Tumor—Rare Disease of the Tricuspid Valves—Sanitary Measures.

EDITORIAL, ---- 86

*Book Notices.*—A Manual of Medical Jurisprudence. By Alfred Swaine Tayor, M.D., F.R.S.

Medical Education—Moses Gunn, A.M., M.D.—Rush Medical College—Settlement of Physicians' Accounts—City Mortality—Proposed New Hospitals upon Randall's Island, New York.

